# Editorial: The mental health impact of weight stigma

**DOI:** 10.3389/fpsyt.2025.1753279

**Published:** 2025-12-26

**Authors:** D. Catherine Walker, Lily O’Hara, Erin N Harrop

**Affiliations:** 1 Union College, Schenectady, NY, United States; 2 Griffith University, Nathan, QL, Australia; 3 University of Denver, Denver, CO, United States

**Keywords:** weight stigma, weight stigma reduction, weight inclusive care, body acceptance, body neutrality

Weight stigma, often referred to as anti-fatness or size-based discrimination, is a deeply ingrained and widespread form of prejudice with profound consequences for mental health and well-being (1, 2). Prevalent in individuals, family relationships, health services, education, employment, social media, legal systems, and all aspects of society, weight stigma creates significant barriers to equitable treatment and quality of life (1). This Research Topic examines the lived experiences, predictors, mediators, and moderators of weight stigma and its mental health consequences, as well as the development, implementation, and evaluation of initiatives to reduce weight stigma and its mental health consequences at the intrapersonal, interpersonal, intersectional, institutional, or ideological levels. We aim to bear witness to the harms of weight stigma, while also exploring practical, creative solutions to mitigate harm, and support individual and communal healing.

Regarding our approach, we aimed to protect against inadvertently perpetuating weight stigma (e.g., through pathologizing weight, reiterating fat stereotypes, treating weight loss as inherently salutogenic, medicalizing “obesity,” “solving” stigma through weight management). To better accomplish this, we provided guidelines for authors on weight-inclusive language that “affirms the respect and human dignity” of those about whom we speak (3). We also aligned our approach to this topic with a weight-inclusive lens (4). The nine articles in this Research Topic that document harms of weight stigma, strategies for healing from weight stigma, and initiatives to reduce weight stigma are briefly reviewed here.

Regarding mental health harms of weight stigma, a survey of U.S. adults found that weight stigma is associated with a range of poor psychological outcomes including lower global mental health scores, more frequent depressive symptoms, and greater odds of depressive disorder diagnosis, which were consistent across racial and ethnic identity, body size, and socioeconomic status (Gerend et al.), indicating that no group is immune to its effects. Another quantitative study with U.S. adults found that perceived stress mediated the relationship between weight stigma and depressive and anxiety symptoms, accounting for 37% of the link (Figueroa et al.), highlighting weight stigma as a potent psychosocial stressor that contributes to psychological distress.

Weight stigma also affects personal relationships, including within families and romantic relationships. A longitudinal study in Spain found that family-based weight stigma negatively impacted adolescents’ psychological well-being, particularly among girls (Anastasiadou et al.). Recent exposure to weight stigma was linked to higher distress, with maternal and paternal comments about weight and dieting associated with increased distress and lower self-esteem, suggesting that educating parents to avoid stigmatizing comments and promote positive, health-oriented, weight-inclusive messages is essential. Romantic relationships are similarly affected. A quantitative study of couples in the U.S. found significant negative associations between an individual’s own internalized and anticipated weight stigma and both their own and their partner’s mental well-being (Brochu et al.). Thus, weight stigma felt by one partner in a couple harms the mental health of both people in the relationship.

Further, weight-based discrimination intersects with other forms of oppression, shaping experiences for those with less access to power and privilege. A qualitative study of sexual minority women in the U.S. found that dominant cultural norms, intergenerational practices within families, and queer communities were all contexts in which weight stigma was both reinforced and resisted (Fowler et al.). This highlights the need to address intersecting forms of oppressions across the lifespan and community contexts, rather than focusing on weight stigma or certain communities in isolation.

Often described as a hostile environment for larger-bodied people, the health system is not immune to weight stigma (Tomlinson et al.). A scoping review found that mental health professionals frequently observed weight bias in colleagues, even if they did not report it in themselves (Philip et al.). Higher-weight clients were perceived more negatively and received less intensive care for similar symptoms than lower-weight clients (Philip et al.). Paradoxically, professionals rated restrictive eating disorder symptoms in higher-weight clients as less severe and recommended less intensive treatment (Philip et al.). Qualitative studies in the review revealed clients’ experiences of weight bias, unsolicited weight loss advice, and differential treatment, which all undermined therapeutic progress and eroded trust in practitioners and the mental health system (Philip et al.).

Turning towards healing, reducing weight stigma requires disrupting traditional paradigms and adopting new strategies, both as individuals and systems. At the individual level, an online survey of racially and ethnically diverse U.S. adults found that those who engaged in adaptive coping, such as cognitive reframing or seeking social support, showed weaker associations between weight stigma and poor mental health outcomes (Gerend et al.), suggesting that more inclusive paradigms and community support could buffer against harms. A case study in a U.S. hospital described the collaborative process of developing a size-inclusive, trauma-informed e-course with separate tracks tailored to clinicians, staff, and patients (Tomlinson et al.), documenting how such paradigmatic shifts and interdisciplinary collaborations happen in the real world.

Online and digital platforms provide effective tools for driving societal change. An online experimental study with women and gender diverse participants in the U.S. found that exposure to brief body positive and body neutral TikTok videos was associated with improved functional appreciation and mood and reduced self-objectification and body dissatisfaction (Kilby and Mickelson). Finally, a qualitative study with US college students with elevated eating disorder psychopathology identified support for a digital adaptation of the Body Advocacy Movement, a program targeting anti-fat bias, as a potential way to buffer eating disorder development (Laboe et al.).

In conclusion, articles in this Research Topic paint a clear picture of weight stigma as a widespread, harmful, intersectional, and complex issue. Weight stigma manifests in families and romantic relationships, influences social interactions across settings, and pervades health services. Its negative effects on mental health are significant, experienced across age, race, sexuality, and other demographic characteristics, often mediated by stress. Reducing weight stigma requires a multi-pronged approach, from educating parents and health service providers to promoting adaptive coping strategies and social support, movements and paradigms that challenge anti-fat bias, promote body acceptance and neutrality, and create size- and weight-inclusive environments.
